# Experimental research on the structural instability mechanism and the effect of multi-echelon support of deep roadways in a kilometre-deep well

**DOI:** 10.1371/journal.pone.0192470

**Published:** 2018-02-15

**Authors:** Rui Peng, Xiangrui Meng, Guangming Zhao, Yingming Li, Jianming Zhu

**Affiliations:** 1 School of Safety Engineering, North China Institute of Science & Technology, Beijing, China; 2 Department of Energy and Security, Anhui University of Science and Technology, Huainan, Anhui Province, China; Massachusetts Institute of Technology, UNITED STATES

## Abstract

We study the structural instability mechanism and effect of a multi-echelon support in very-deep roadways. We conduct a scale model test for analysing the structural failure mechanism and the effect of multi-echelon support of roadways under high horizontal stress. Mechanical bearing structures are classified according to their secondary stress distribution and the strength degradation of the surrounding rock after roadway excavation. A new method is proposed by partitioning the mechanical bearing structure of the surrounding rock into weak, key and main coupling bearing stratums. In the surrounding rock, the main bearing stratum is the plastic reshaping and flowing area. The weak bearing stratum is the peeling layer or the caving part. And the key bearing stratum is the shearing and yielding area. The structural fracture mechanism of roadways is considered in analysing the bearing structure instability of the surrounding rock, and multi-echelon support that considers the structural characteristics of roadway bearings is proposed. Results of the experimental study indicate that horizontal pressure seriously influences the stability of the surrounding rock, as indicated by extension of the weak bearing area and the transfer of the main and key bearing zones. The falling roof, rib spalling, and floor heave indicate the decline of the bearing capacity of surrounding rock, thereby causing roadway structural instability. Multi-echelon support is proposed according to the mechanical bearing structure of the surrounding rock without support. The redesigned support can reduce the scope of the weak bearing area and limit the transfer of the main and key bearing areas. Consequently, kilometre-deep roadway disasters, such as wedge roof caving, floor heave, and rib spalling, can be avoided to a certain degree, and plastic flow in the surrounding rock is relieved. The adverse effect of horizontal stress on the vault, spandrel and arch foot decreases. The stability of the soft rock surrounding the roadways is maintained.

## 1. Introduction

The gradual development of the Chinese mining industry has now entered the kilometre-deep mining stage. Then scholars employ classical elastic–plastic mechanics on surrounding rock to analyse the instability of shallow roadways. Examples of these approaches include the elastic–plastic mechanics of crack extension[1] and the elastic–plastic stress field in cracked bodies [2]. As a mine enters the deep excavation stage, examining the strength degradation and secondary stress distribution of surrounding rock becomes increasingly difficult due to the increases in horizontal stress induced after deep cracked roadway excavation. A number of scholars have proposed complex methods for analysis, such as the modified Kastner formula for cylindrical cavity contraction [3] [4], the elastic–plastic solution to the analysis of the deep tunnel under water [5] [6], and the deep soft roadway failure modes and evolution process [7].

The instability of deep roadways often results from the structural fracture [8], which cannot be sufficiently explained in the light of the classical elastic–plastic mechanical analysis method for surrounding rock [9]. Therefore, problems with roadway instability resulted from deep excavation cannot be easily understood, constituting a hindrance to the design for surrounding rocks using the effective support mechanism. Deep roadways are frequently in need of repairs for the instability and the repair will lead to the immense economic loss.

**Fig 1:**
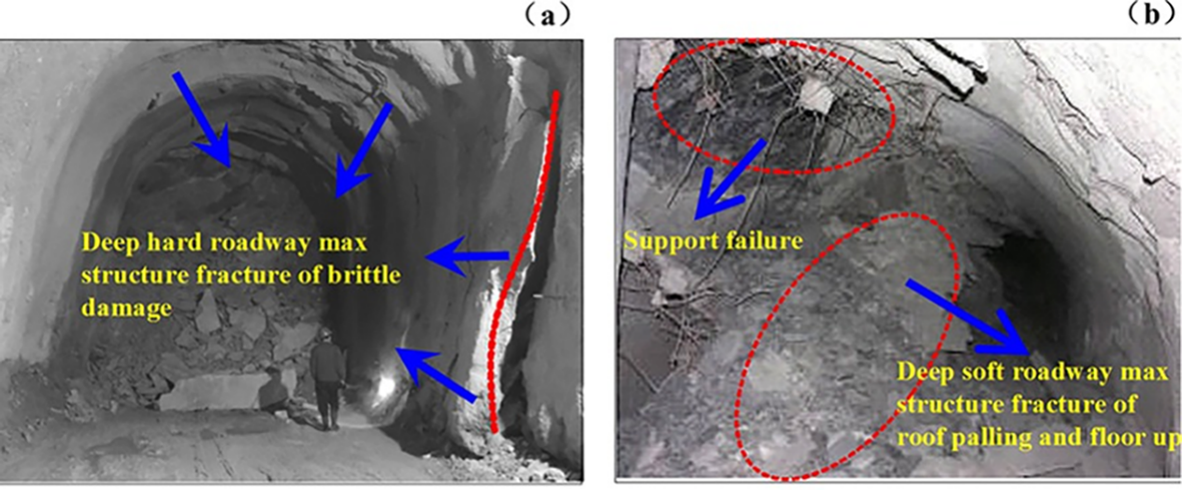
Maximum structural fracture of (a) deep hard roadway; and (b) deep soft roadway.

The high horizontal stress in depth can give rise to the serious roadway deformation, leading to the support failure and the instability of the bearing structure. For our purpose, a Huaibei mine 1000m in depth was selected. For the experiment, two fairly unstable soft rock tunneling roadways were selected. The maximum structural fracture and support failure of deep roadway after excavation are shown in Fig 1.

Scholars have proposed various bearing structures based on the strength degradation and secondary stress distribution of surrounding rock. Examples of the proposed structures include the loss bearing circle [9][10][11], the key bearing circle by Kang [12][13], the main and subordinate bearing circles [14], the internal and external bearing circles[15], the coupling arch bearing structure[16], and the continuous double crust[17].

Whether by means of theoretic deducing or experimentation, the above-mentioned researchers all believe that it is necessary to partition a roadway into different bearing stratums but they are different only in partition methods and rationale. However, they have failed to consider factors such as non-uniform stress field, shear failure, and the relationship between mechanical bearing structure and quantitative support design of the surrounding rock.

Traditional support designs for shallow roadways provide support strength by employing the combined support with anchoring, net, spraying, anchor cable and grouting. This approach, which works by reducing row distance, is called the intensive support method. The instability of deep excavation roadways cannot be addressed using empirical support without theoretical basis. Various studies have been conducted on coal roadway support designs, particularly multi-echelon support [18][19][20], which provides support for coupling roof coal roadways and can be used in deep rock roadways.

This study therefore proposes the coupling mechanical bearing structure and multi-echelon support principle according to in-situ measured data. A scale model test scheme can be designed according to the prototype. The experimental analysis of the mechanical bearing structure without support is influenced by the confining pressure in the non-uniform stress field. Thus, multi-echelon support is proposed, and the stability of the mechanical bearing structure with such a support is analysed. Moreover, the effect of the proposed multi-echelon support on the stability of deep excavation roadways is determined by examining the rupture degree of rock and surveying the loose circle by in-situ and numerical simulation.

## 2. Engineering geological data of the original kilometer-deep mine

**Fig 2:**
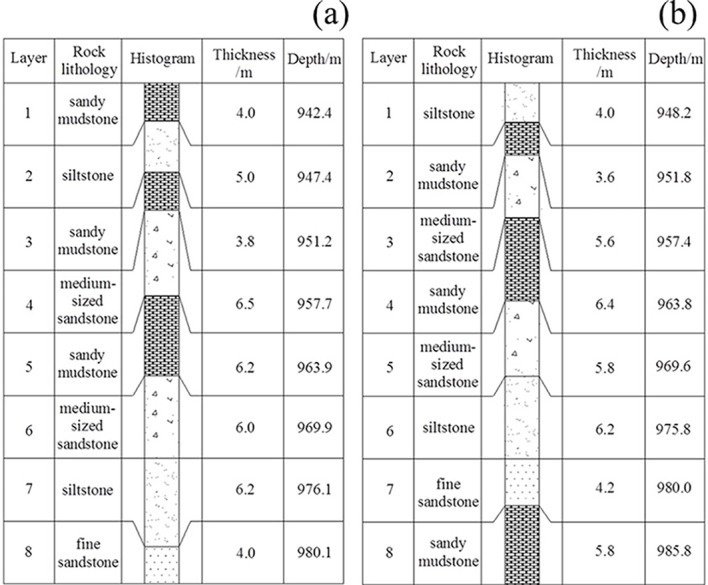
Drilling histogram near (a) cross point 1; and (b) cross point 2.

According to the in-situ exploration datum of a kilometre-deep mine, the two cross point roadways with poor stability are named as the cross point 1 and cross point 2, which have level elevations of -955m and 960m, respectively. The lithology of the surrounding rock in the cross point 1 and cross point 2 are high-strength medium-sized sandy and low-strength sandy mudstone, respectively. The drilling histogram of each cross point is illustrated in Fig 2.

**Fig 3:**
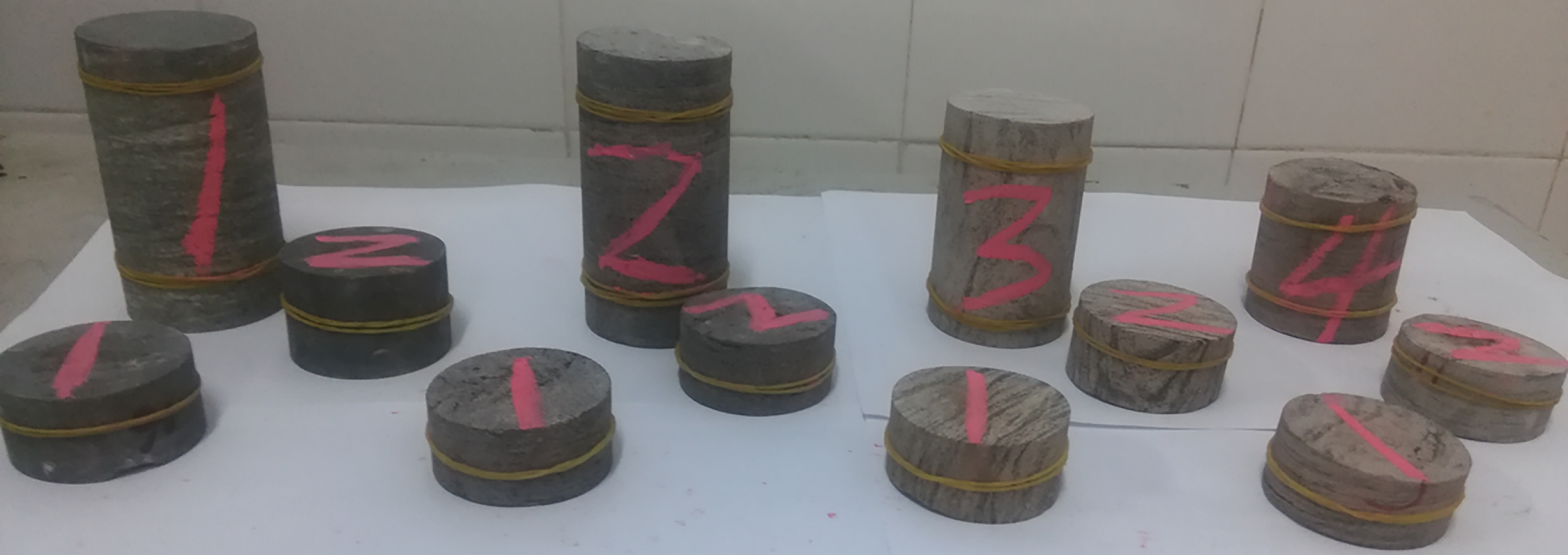
In-situ rock samples.

The in-situ rock samples are drilled out from different cross points in deep mine, which are listed in Fig 3. Section numbers 1–4 correspond to sandy mudstone, siltstone, medium course sandstone, and fine sandstone, respectively.

The mechanical parameters of each type rock, are tested by MTS uniaxial compress test, and are listed in Table 1.

**Table 1:** Strength of rock samples at different cross points.

Rock lithology	Elasticity modulus/GPa	Deformation modulus/GPa	Compressive strength/MPa	Shear strength /MPa
Fine mudstone	20.06	22.13	98.10	12.15
Medium course sandstone	16.06	17.34	75.10	11.73
Sandy mudstone	7.19	8.65	35.06	7.84
Siltstone	5.56	6.42	25.02	4.17

## 3. Coupling bearing structure of surrounding rock and multi-echelon support

### 3.1. Dividing theory basis of coupling bearing structure of surrounding rock

Before excavation, the surrounding rock in the tunnel remained in the elastic state and so it was rather stable. In the excavation, the stress was redistributed, hence the formation of the mechanic bearing structure. At the moment, the ring stress varied most sensitively and the variation trend approached the full stress-strain curve of the rock. Taking this into account, based on the ring stress concentration degree, the bearing structure of the surrounding rock was discussed and the discussion was rather convincing. It would be convenient to describe the rock roadway by means of polar coordinates. Therefore, the maximal, medial and minimal major stresses *σ*_1_, *σ*_2_ and *σ*_3_ could be expressed by the ring, axial and radial stresses *σ*_*θ*_, *σ*_*z*_ and *σ*_*r*_ in the polar coordinates.

The shearing damage to the surrounding rock is frequently recognized in rock mechanics and engineering researches. But with three dimensional stresses involved, it is difficult to deduce the shearing stress of the surrounding rock. For this reason, they usually turn to the following means. First, the concept of equivalent stress is introduced. Then under complicated stress conditions, the equivalent shear stress is calculated. Finally, by taking into account the secondary stress distribution and the strength deterioration of the surrounding rock, the mechanical bearing structure of the surrounding rock can be rather accurately partitioned.

The roadway mechanical model changes from a 3-D model to a 2-D model after excavation and damage, that is the problem of thin-walled circular plate in rock mechanics underground, which can be treated as a plane strain model[21][22]. In the plane strain state, the equivalent stress *σ*_i_ in complex stress condition can be simplified as

$$\sigma_{i}=\sqrt{{\left(\sigma_{\theta }-\sigma_{r}\right)}^{2}+{\left(\sigma_{r}-\sigma_{z}\right)}^{2}+{\left(\sigma_{z}-\sigma_{\theta }\right)}^{2}}/\sqrt{2}=\sqrt{\sigma_{\theta }^{2}+\sigma_{r}^{2}-\sigma_{\theta }\sigma_{r}}$$ (1)

Where *σ*_z_ = 0.

Based on the relationship between equivalent stress and equivalent shear stress and combined with Eq (1), the equivalent shear stress in complex stress condition can be written as

$$\tau_{i}=\frac{\sigma_{i}}{\sqrt{3}}=\frac{\sqrt{\sigma_{\theta }^{2}+\sigma_{r}^{2}-\sigma_{\theta }\sigma_{r}}}{\sqrt{3}}$$ (2)

Where *τ*_*i*_ is the equivalent shear stress.

According to the generalized Hooke’s law,

$$\frac{\left(1+\nu \right)\left(\sigma_{\theta }-\sigma_{r}\right)}{E}=\varepsilon_{\theta }-\varepsilon_{r}$$ (3)

Combined with the equilibrium differential equation,

$$\frac{d\sigma_{r}}{\mathrm{dr}}-\frac{\sigma_{\theta }-\sigma_{r}}{r}=0$$ (4)

By obtaining corresponding tangential and radial stress,

$$\left\{ \begin{array}{l} \sigma_{\theta }=\frac{E}{1+\nu }\left(\varepsilon_{\theta }-\varepsilon_{r}\right)+\sigma_{r}\\ \sigma_{r}=r^{\frac{E}{1+\nu }\left(\varepsilon_{\theta }-\varepsilon_{r}\right)}e^{-3.27} \end{array}\right.$$ (5)

Finally, the surrounding rock can be accurately classified as a macro coupling mechanical bearing structure when the secondary stress distribution and strength degradation of the surrounding rock are considered.

### 3.2. Dividing method of the coupling mechanical bearing structure of surrounding rock

Scholars have neglected the influence of rock lithology and shear stress distribution on stress-bearing structures in past studies. This paper proposes a new stress-bearing structure based on different lithologies. According to the subsequent test results, a 1.1-fold in situ stress (proposed by Li, 2006) is appropriate for weak bearing areas in soft roadways. However, this value is relatively low for hard roadways, considering that the weak bearing area should be located in roofs, sides, and arch floors. A 1.5-fold in situ stress (proposed by Kang, 1997) is inappropriate for the main bearing area in soft and hard roadways. Thus, the 1.2-fold and 1.3-fold are proposed in this paper, respectively, considering that the outline of the main bearing area should be located in the plastic area and its depth should be greater than that of the key bearing area. The concentrated equivalent shear stress is the basis for classifying the key bearing area, which should be higher than the shear yield stress of different rock lithologies.

1. The weak bearing area is the initial bearing area with a weak bearing capacity. In soft rock roadways, this bearing area mainly exists in the fracture area and soft area, the tangential stress concentration is in the range of 0 to 1. 1*p*_0_. In hard roadways, this bearing area mainly exists in the damaged area, where the stress concentration is in the range of 0 to 1.2 *p*_0_, which is regarded as a loose circle in designing the initial support. This bearing area found itself close to the fractured rock surrounding roadway wall. It could not offer a strong bearing force and was in need of spraying support to reinforce the strength of the surrounding rock in order to prevent waste from dropping or roof from caving. And it is a stratum which exercises rather great impact on the stability of the bearing structure of the roadway.2. The key bearing area is the area where shear stress is concentrated. This bearing area was a stratum crucial to the balance of the whole bearing structure and it found itself in the softening area (or damaged area). If the shearing pressure should induce damage, the weak bearing stratum would move within the bearing structure. In this case, it is necessary to reinforce the shearing resistance strength by dense bolting and spraying.

3. The main bearing area is the area where tangential stress is concentrated. The concentration coefficients in the soft rock and hard rock are higher than those under 1.2-fold in-situ stress and 1.3-fold in-situ stress, respectively. The bearing capacity of this area is strong and mainly exists in the soft area (or damaged) and elastic area. With the excavation of the roadway, it would move internally and the range would be extended, not beneficial to the stability at the initial stage of the tunneling. For this reason, during the excavation, engineers managed to keep the stratum as close to the roadway wall as possible. To realize the goal, the support was reinforced to prevent damage to the roadway and stop the internal movement of the peak value.

**Fig 4:**
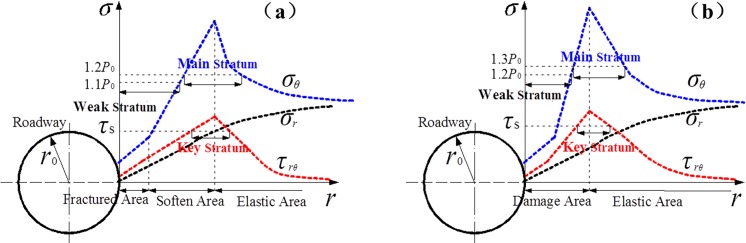
Mechanical structure model of (a) cross point 1; and (b) cross point 2.

The coupling mechanical bearing structure combined with the surrounding rock mechanical model is depicted in Fig 4.

### 3.3. Multi-echelon support design theory

The traditional and empirical support method for deep rock roadways is the combined support with anchoring, net, sprayed concrete, anchor cable, and grouting [20]. The empirical method, which reduces the inter-row spacing of the support, fails to efficiently address the destabilisation problem of deep soft or hard roadways. Various studies on deep coal roadway support have been conducted. The instance is taken considering the coupling surrounding rock. Multi-echelon support[18][19]is always used in coupling soft roof of deep coal roadways.

If the coupling mechanical bearing structure mentioned above can be considered as coupling surrounding rock, then the rock roadway support can be designed as a multi-echelon support based on coupling soft roof of coal roadways. Thus, the traditional and empirical support method for rock roadways can be redesigned as follows.

The first layer which is building shell support, is the combined support with anchoring net, spraying and grouting of the weak bearing structures. This layer prevents the roof falling and fractured surrounding rock. An effective support range of anchoring and grouting is designed according to the outer boundary of the weak bearing structures. The grouting is a full-length grouting. The thickness of spraying can be designed according to the weak bearing structure range and fracture degree of surrounding rock.

The secondary layer, which is anchoring and end grouting support, supports the key bearing structure, the effect range of end grouting is designed according to the key bearing area, and end grouting can enhance the shear strength of this bearing area.

The third layer, short anchor cable suspension support, supports the main bearing area. The strength of the surrounding rock in this bearing area is high. This bearing area is set as the foundation of the entire bearing structure. The short cable length is designed according to the outer boundary of the main bearing structure.

**Fig 5:**
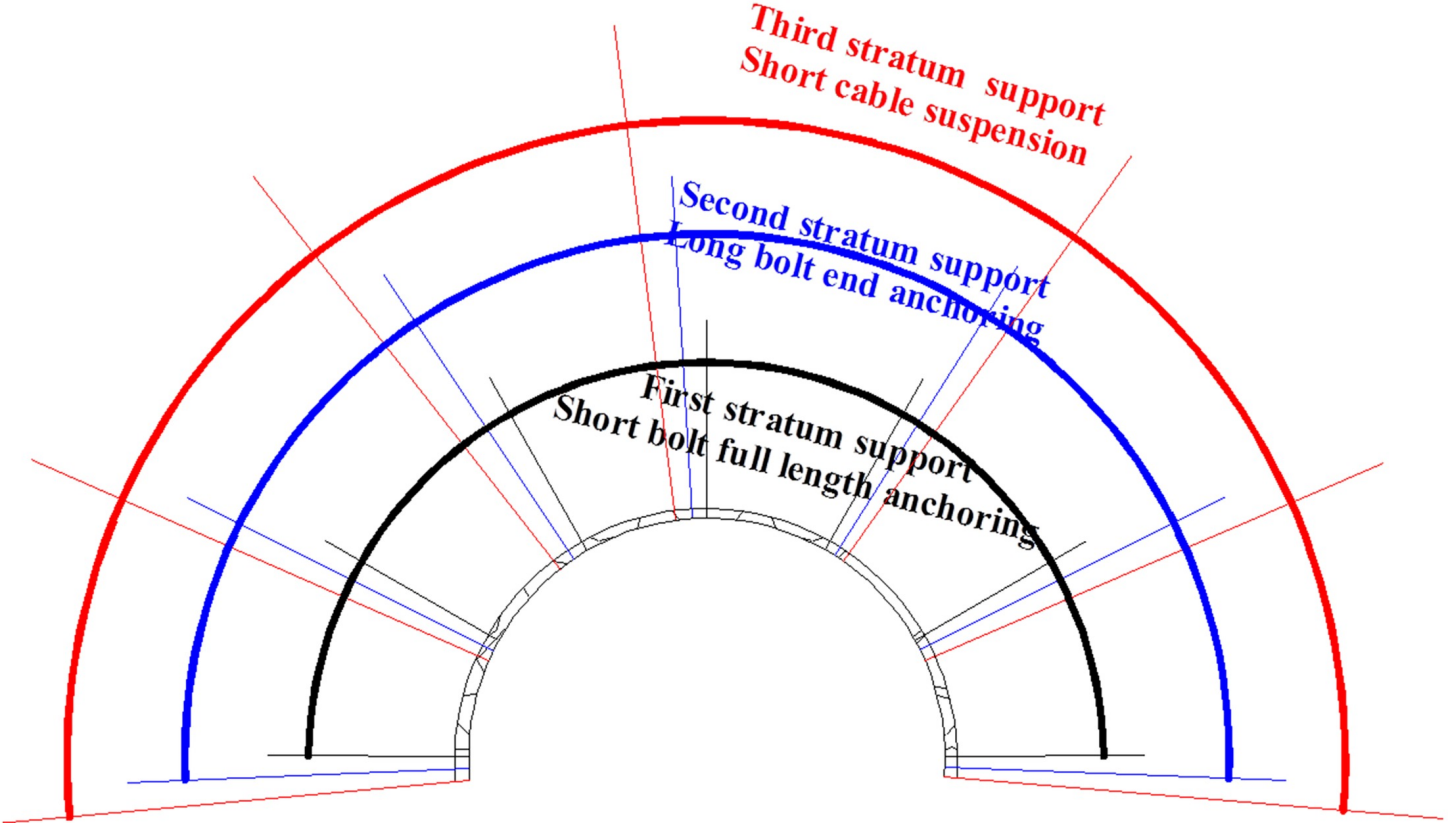
Multi-echelon support of surrounding rock.

The improved support process can be summarised as a multi-echelon support comprising full length grouting and end grouting and anchor cable suspension. The scheme is depicted in Fig 5.

## 4. Design of scale model test scheme

### 4.1. Scale factor design and test bench assemble

1. Geometric scale factor: The tunnel prototype radius is 2.5 m, and the boundary prototype is 45 m × 30 m × 7.5 m. These dimensions are selected to avoid the sizing effect. The boundary prototype that corresponds to the model size is 1.8 m × 1.2 m × 0.3 m. The geometric scale factor is *C*_*L*_ = 1: 25.
2. Unit weight scale factor: The physical mechanical parameter test of the in-situ and similar standard specimen indicate that the unit weight scale factor is *C*_*γ*_ = 1:1.5.
3. The stress scale factor is *C*_*σ*_ = *C*_*L*_ × *C*_*γ*_ = 1:37.5.
4. Jack load calculation: The lateral baffle area of the model is 120 cm × 30 cm = 3600 cm^2^. The total area of the two pistons on each side of the jack is 56 cm^2^. Thus, the scale factor of the horizontal jack and boundary load is 64.286:1. The roof area is 180 cm × 30 cm = 5400 cm^2^, and the total area of the two pistons in the roof is 56 cm^2^. Thus, the scale factor of the vertical jack and boundary load is 96.43:1.

**Fig 6:**
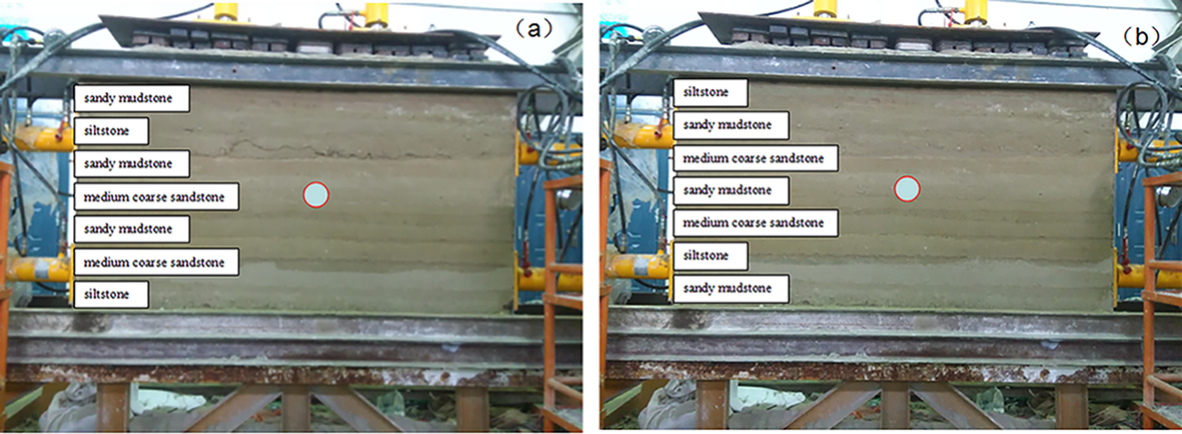
Plane strain test rig model of (a) cross point 1; and (b) cross point 2.

The plane strain rig is assembled into loading device using a jack. The device is assembled into a plane counter-force experiment device, comprising a plane model rig, jack, flange, and high-strength bolt. The jack and flange are fixed on the upper shelf and two sides of the model by the high-strength bolt. The size of the experiment rig is 3 m × 2 m × 0.3 m, while the size of the test model size is 1.8 m × 1.2m × 0.3 m. The simulated roadway radius is 12 cm. The scale model test device is presented in Fig 6.

### 4.2. Test of similar material mechanical properties

The mechanical tests for similar material are conducted on three groups. The scale factors of the aggregate material (sand), agglutinate material (lime and cement), and water in each test group of similar materials are conducted as 2:1:0.2, 4:1:0.4, 6:1:0.6, 8:1:0.8, and 10:1:1. The scale factor of sand and water is maintained at 10:1.

A similar material in a comparable scale factor is placed in a cylindrical standard vessel and compacted to the standard specimen with an appropriate height (over 110 mm).

**Fig 7:**
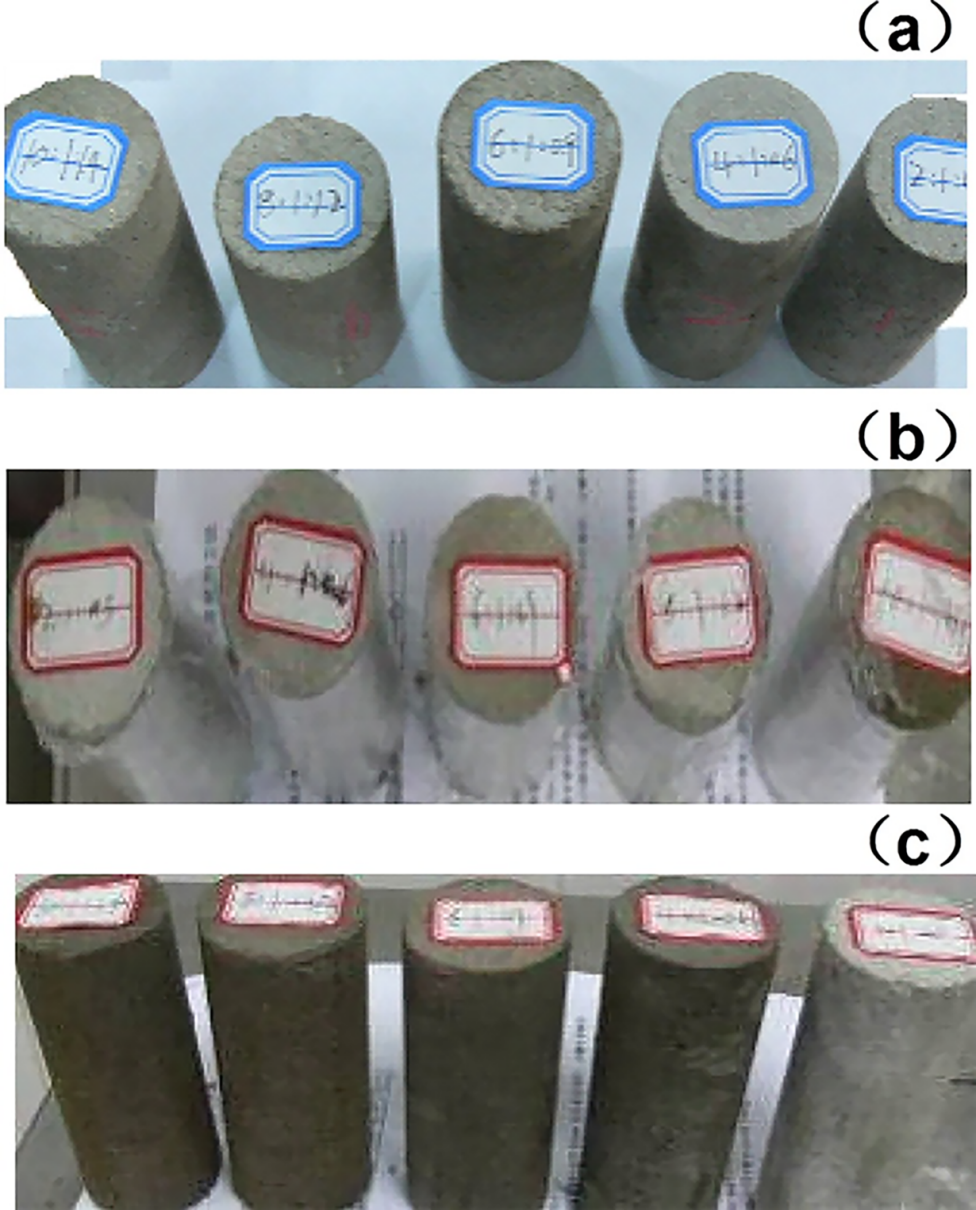
Similar material standard rock samples of the (a) first group; (b) second group; and (c) third group.

The standard specimens are labelled based on their classifications and placed in a laboratory, as shown in Fig 7. After a certain age (21 days) is reached after the grinding process, the strength parameters are tested in the RMT mechanical testing machine. The experimental results are shown in Table 2.

**Table 2:** Mechanical parameters of the similar material.

Group name	Rock samples mechanical parameters	Scale factor of sand, solidified material and water				
		2:1:0.2	4:1:0.4	6:1:0.6	8:1:0.8	10:1:1
First group	Compressive strength/MPa	5.103	3.126	1.711	1.029	0.733
	Elasticity modulus/GPa	2.070	0.815	0.366	8.107	0.577
	Deformation modulus/GPa	2.114	1.401	1.392	19.24	3.013
	Shear strength/MPa	1.412	0.425	0.362	0.331	0.225
Second group	Compressive strength/MPa	5.215	3.015	1.721	1.108	0.743
	Elasticity modulus/GPa	2.133	0.926	0.358	8.215	0.589
	Deformation modulus/GPa	2.238	1.512	1.403	20.01	3.120
	Shear strength/MPa	1.542	0.531	0.384	0.341	0.232
Third group	Compressive strength/MPa	4.986	2.853	1.701	1.029	0.713
	Elasticity modulus/GPa	1.975	0.801	0.343	8.012	0.551
	Deformation modulus/GPa	2.012	1.395	1.380	19.14	2.987
	Shear strength/MPa	1.398	0.418	0.354	0.320	0.210

The experimental results indicate that the scale factor of the aggregate material and cement within a certain range, rock compressive strength, and shear strength decreases as the cement content decreases. The in-situ rock strengths of fine sandstone, medium course sandstone, sandy mudstone, and siltstone are approximately 98, 75, 35, and 25 MPa, respectively, considering that the stress scale factor is 1:37.5. The corresponding similar material strengths are 2.613, 1.86, 0.933, and 0.667 MPa, respectively. Based on the loading ranged within 1MPa by taking into account the similar rock strength, the similar material matching scale factors selected were 4:1:0.4, 6:1:0.4, 8:1:0.8, and 10:1:1, respectively. The corresponding average compressive strengths are 2.998, 1.711, 1.029, and 0.733 MPa, respectively, sufficient to satisfy the demand for the partition of the roadway bearing structure and the observation of the roadway deformation and fracture development.

### 4.3. Roadway model making and strain gauge setting without support

**Fig 8:**
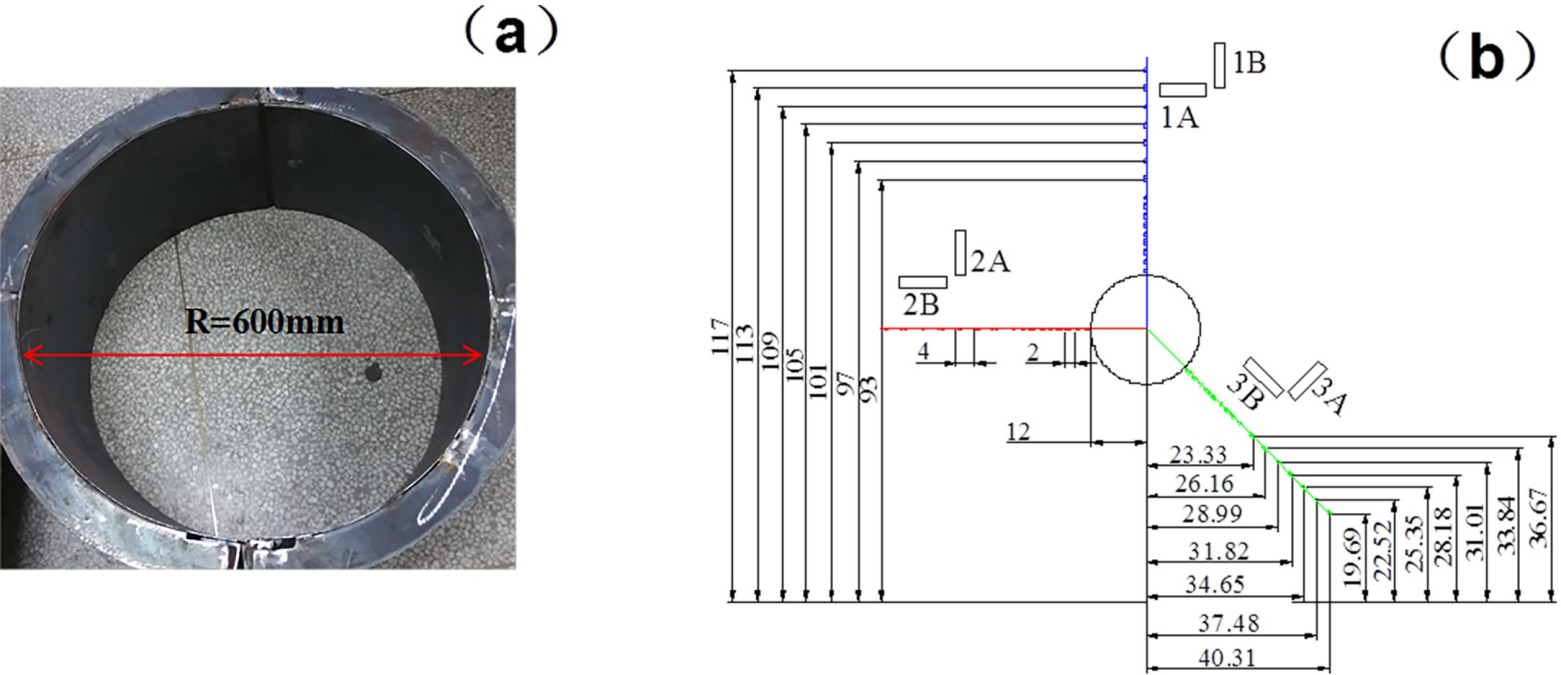
Figure about (a) making roadway model; and (b) laying strain gauge.

To facilitate sticking strain gauges along the lines in various directions, a roadway model should be prepared. As shown in Fig 8(A), the diameter of the roadway model was 600mm. The survey lines for testing the surrounding rock stress are set along the roadway roof, side, and arch foot. Strain gauges are pasted along the radial and tangential directions using a strain brick. The gauges are set, as shown in Fig 8(B). The strain brick material is consistent with the roadway material. The excavation roadway radius is 12 cm and the external surrounding rock is 18 cm within the roadway model.

Inside the roadway model, nine measuring points are laid along each survey line around the excavation roadway. The distance of each measuring point is 20 mm. Each point contains two strain gauges in both radial and tangential directions. Outside the roadway model, seven measuring points are laid along each survey line on the experiment rig at 40 mm intervals. Each survey line contains 16 measure points on the entire experiment rig. The layout principle is shown in Fig 8(B), where each ID number is presented in centimetres. The labels of 1A, 2A and 3A indicate the tangential direction, and the labels of 1B, 2B and 3B represent the radial direction.

Before the roadway model is placed on the experiment rig, epoxy resin is smeared on the outer wall of the roadway model of the surrounding rock, and similar material is dumped in the experiment rig, which is consistent with the mechanical properties of the surrounding rock in roadway model. Then a load was imposed horizontally and vertically so that the model and the surrounding should be closely contacted so as to avoid the adverse impacts on the strain by the model.

### 4.4. Constructing roadway model and setting strain gauge under multi-echelon support

**Fig 9:**
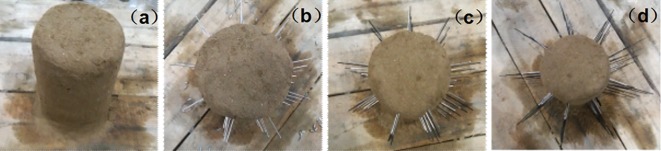
Multi-echelon support design of (a) excavation roadway; (b) first-layer support; (c) second-layer support; and (d) third-layer support.

Multi-echelon support is designed according to the characteristics of the mechanical bearing structure of the surrounding rock without support. The excavation roadway is shown in Fig 9(A). The three layers of multi-echelon support include the first layer full length grouting support, the second layer key bearing area end grouting support, and the third layer short cable suspension support. The layers are presented respectively in Fig 9(B), 9(C) and 9(D). The anchoring and anchor cable are shown in Fig 9. The anchor is made of soft aluminium alloy material, and there is a thread on its stern. After roadway excavation, the anchor bolt and cable are attached closely to the roadway surrounding rock by tightening the soft aluminium alloy material with the nut, which can provide a certain pre-tightening force to bear the load of the surrounding rock. Then we apply the tension force to modeled anchor by tightening the anchor with the nut, and maintain that the anchoring is not loosened with a certain tension force. That is the tension force provided by pre-tightening force anchor, but it cannot control the value of the tension force by the soft aluminium alloy material.

**Fig 10:**
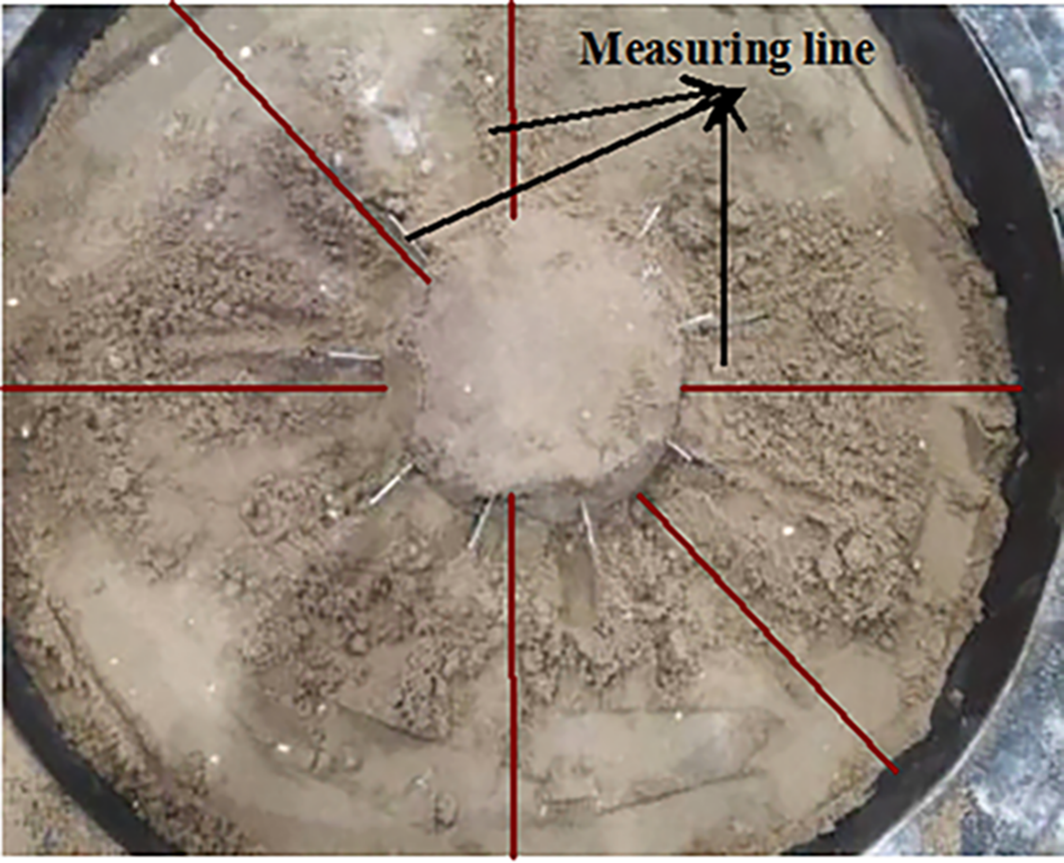
Multi-echelon support and strain gauge layout.

The excavation roadway and its surrounding rock are made by pouring concrete in a roadway mould. To test the secondary stress of the surrounding rock, strain gauges are pasted in the radial and tangential direction using strain bricks along the measure lines in the roadway roof, side, and arch foot.Then materials were put into the roadway model which should be in agreement with the entire model in terms of surrounding rock mechanical property. The entire model is described in Fig 10.

### 4.5. Experiment scheme design

Different groups are controlled in the experiment. The first group scale model test without support is simulated under the influence of different confining pressures on the bearing structure. The materials are first loaded before excavation. The specific processes are listed as follows. In the scale model experiment, the buried depths are -955 and -960 m, which correspond to the ground pressures of 23.87 and 24.00 MPa, respectively.

After roadway excavation without support, the surrounding strain values of each measure line of the roadway are measured under different confining pressures by changing the lateral pressure coefficient. The secondary stress field is calculated based on the measured strain value, and the stress bearing structure is divided.

Based on scale factor of loading stress and the conversion relationship between the jack load and model boundary force, the scale factor of the single horizontal jack and boundary load is 32.143:1. The scale factor of the single vertical jack and boundary load is 48.215:1. The loading stress of jack in different cross points is listed in Table 3.

**Table 3:** Loading stress of jack in different cross points.

Horizontal pressure coefficient			λ = 1	λ = 1.5	λ = 2
Cross point 1	In-situ stress/MPa	Vertical	23.870	23.870	23.870
		Horizontal	23.870	35.805	47.740
	Jack stress/MPa	Vertical	61.381	61.381	61.381
		Horizontal	40.920	61.380	81.840
Cross point 2	In-situ stress/MPa	Vertical	24.000	24.000	24.000
		Horizontal	24.000	36.000	48.000
	Jack stress/MPa	Vertical	61.715	61.715	61.715
		Horizontal	41.143	61.715	82.286

In the second group scale model test, multi-echelon support is designed according to the bearing structure of the surrounding rock in the first group scale model test. The influence of confining pressure on the bearing structure is also simulated.

## 5. Specific experiment operation and data analysis

The influence of confining pressure and support on the stability of the bearing structure is analysed based on the measurement of surrounding rock stress variation and the observed development of the fracture rock. The result indicates that the instability of the bearing structure is the major factor that affects the structural fracture of deep roadways. Then the effect of multi-echelon support is examined.

### 5.1. Structural stability analysis of surrounding rock without support

By taking the cross point 1 as an example, the mechanical parameters of the surrounding rock are obtained in the direction of the floor arch foot at the first measured point by changing the lateral pressure coefficient. The calculation methods are listed as follows:

1. In the uniform stress field, the jack loads in the horizontal and vertical directions are 40.920 and 61.381 MPa, respectively, after the layout on each measure line is completed. The tangential and radial strains are 0.670 and 0.291, respectively, at the first measure point, and are determined by the strain gauge on the floor arch foot measure line., The tangential stress and radial stress are 0.307 and 0.133 MPa, respectively, based on the similar material elastic modulus E = 0.458 MPa of the medium course sandstone. When a stress scale factor *C*_*σ*_ = 1:37.5 is applied, the tangential stress and radial stress are 11.5 and 5.0 MPa, respectively. The equivalent shear stress is 5.77 MPa based on Formula (2).
2. The lateral pressure coefficient is 1.5 in the non-uniform field. Both jack loads in the horizontal and vertical directions are 61.381MPa, and the experimental conditions are retained. The authentic tangential stress, radial stress, and equivalent shear stress are 13.5, 5.8, and 6.9 MPa, respectively.
3. The lateral pressure coefficient is 2 in the non-uniform field. The jack loads in the horizontal and vertical directions are 81.840 and 61.381 MPa, respectively, and the experimental conditions are retained. The authentic tangential stress, radial stress, and equivalent shear stress are 14.3, 6.3, and 7.3 MPa, respectively.

**Fig 11:**
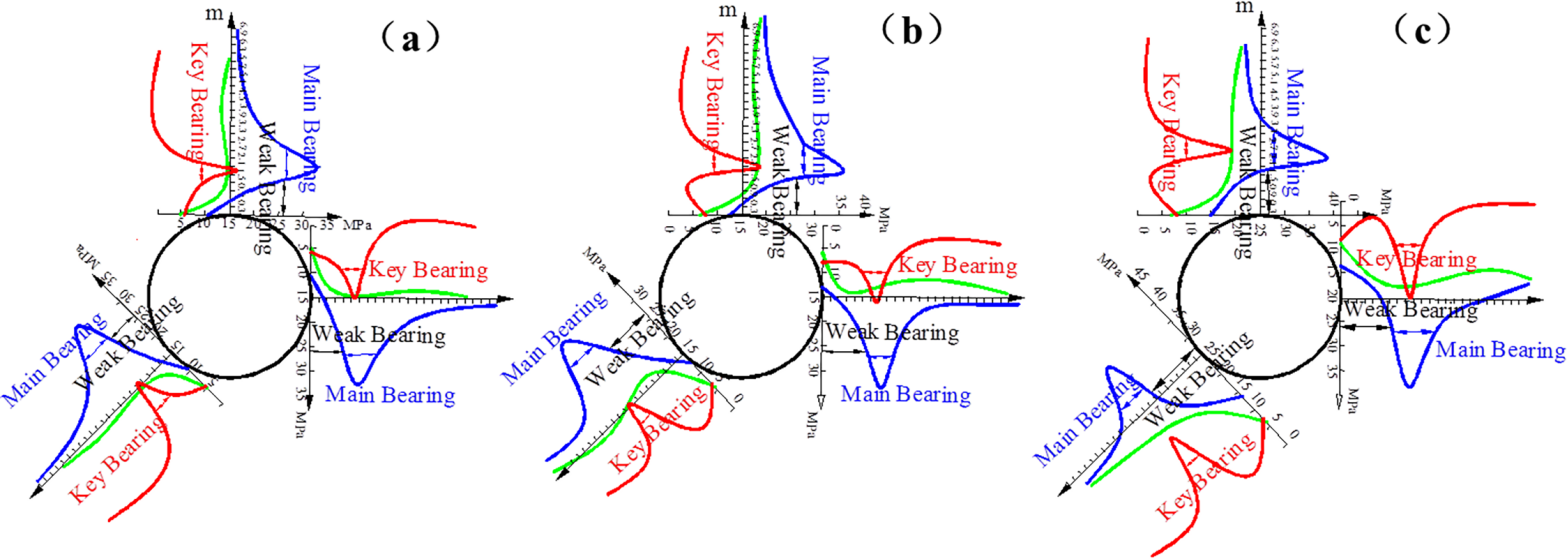
Bearing structure in cross point 1 without support when the horizontal press coefficient is (a) *λ* = 1; (b) *λ* = 1.5; and (c) *λ* = 2.

**Fig 12:**
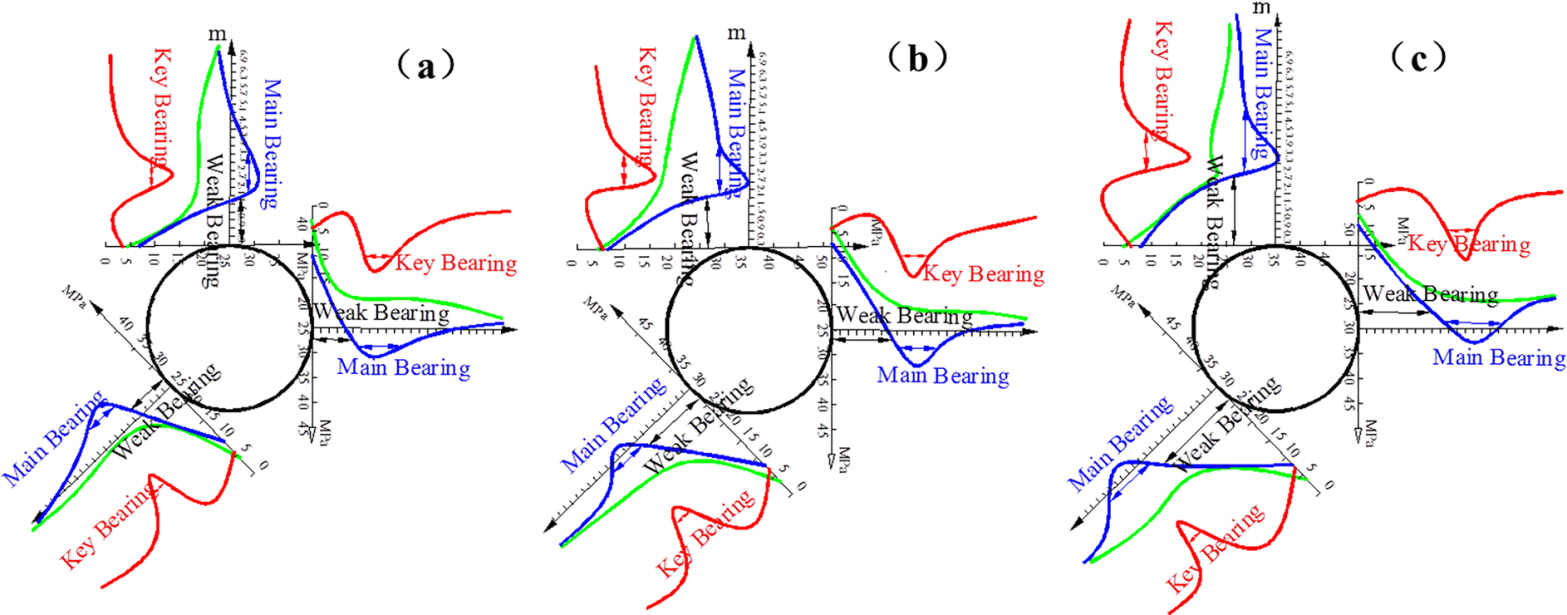
Bearing structure in cross point 2 without support when the horizontal press coefficient is (a) *λ* = 1; (b) *λ* = 1.5; and (c) *λ* = 2.

The secondary stress distributions are obtained in the direction of the roof, sides, and bottom arch foot measure lines according to the above operational process and the processing method. The coupling bearing structure of the surrounding rock is divided. The effects of confining pressure on the coupling bearing structure in the cross points 1 and 2 are obtained, as indicated in Figs 11 and 12, respectively.

The blue, green, and red curves correspond to the distribution of tangential stress, radial stress, and equivalent shear stress, respectively, as shown in Figs 11 and 12. The locations of the weak, key and main bearing areas are likewise presented in Figs 11 and 12.

Figs 11 and 12 show that when the confining pressure is kept constant, the cross point 2 is comparable with the cross point 1. The entire bearing structure moves to the outside, and each bearing area is extended. For example, the weak bearing area expands the range, the main and key bearing areas transfer to the deep radial surrounding rock, the areas of rupture and plastic flow increase, the bearing capacity of the bearing zone declines, the difficulty of the support increases, and the instability of the surrounding rock worsens.

In the same test point, the decrease of the bearing capacity at the two sides and the bottom arch foot is apparent as the lateral pressure coefficient increases. The thickness values of the weak, key and main bearing areas in the direction of the roof, sides, and bottom arch foot are listed in Table 4.

**Table 4:** Thickness of bearing structure without support.

Cross points	Thickness/m	*λ* = 1	*λ* = 1.5			*λ* = 2		
			Roof	Side	Arch foot	Roof	Side	Arch foot
Cross point 1	Weak bearing area	1.30	1.45	1.50	1.80	1.65	1.95	2.40
	Key bearing area	2.00	2.35	2.55	2.85	2.85	3.05	3.40
	Main bearing area	2.60	2.60	2.80	3.35	3.15	3.50	3.65
Cross point 2	Weak bearing area	1.75	1.95	1.90	2.55	2.55	2.70	3.30
	Key bearing area	3.05	3.50	3.20	3.85	4.15	4.35	4.80
	Main bearing area	3.45	3.80	3.70	4.25	5.10	5.40	5.90

### 5.2. Effect of multi-echelon support on bearing structure stability

**Fig 13:**
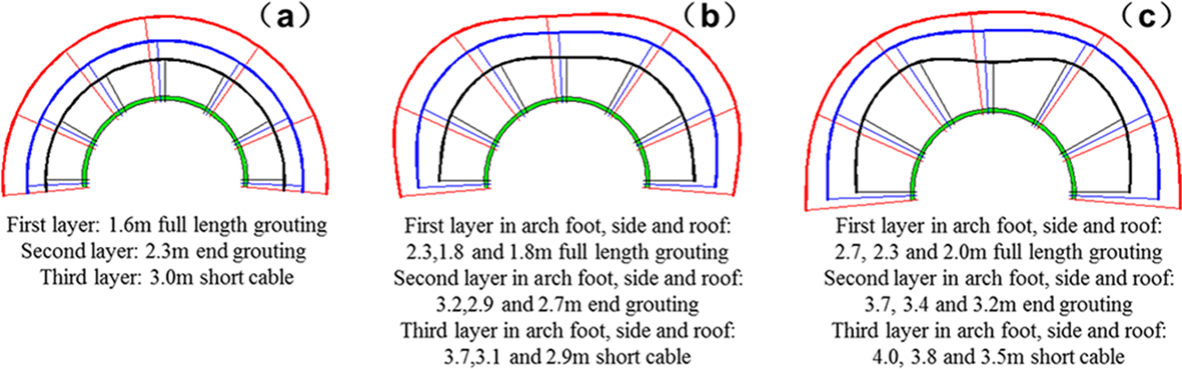
Multi-echelon support design of the cross point 1 when the horizontal press coefficient is (a) *λ* = 1; (b) *λ* = 1.5; and (c) *λ* = 2.

**Fig 14:**
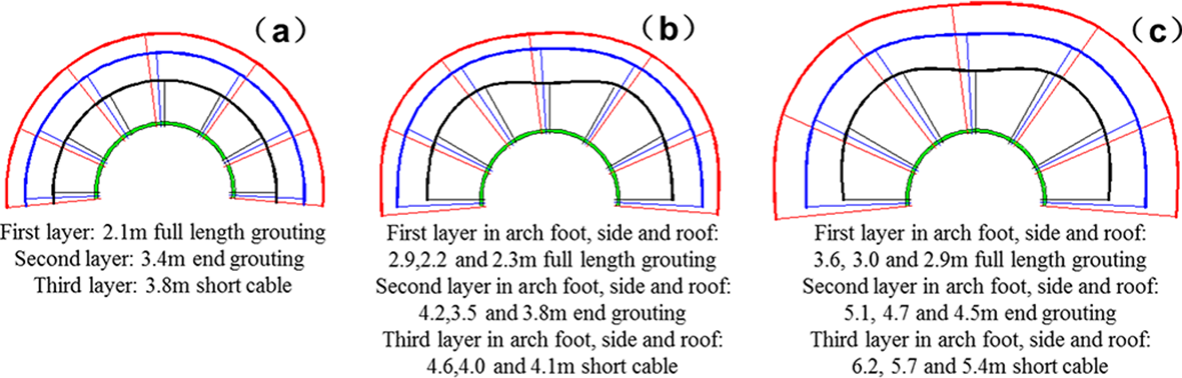
Multi-echelon support design of the cross point 2 when the horizontal press coefficient is (a) *λ* = 1; (b) *λ* = 1.5; and (c) *λ* = 2.

Multi-echelon support is designed according to the outer boundaries of the bearing zone without support as listed in Table 4. The principle of multi-echelon support and its parameters are shown in Figs 13 and 14. The black, blue and red areas correspond to the first-, second- and third-layer supports, respectively. To design the length of anchor bolt and cable, the following parameter must be considered that the spraying thickness is 300 mm. The inter-row spacing of the anchor bolt and cable is 1600mm × 1600 mm. Each support layer must be staggered at a certain distance between the anchor bolt and cable. The actual roadway is an axial-symmetry roadway. Thus, only the support design of the semicircle roadway is provided. The multi-echelon support of the arch foot corresponds to that of the arch shoulder.

The design of multi-echelon support in different cross points under different lateral pressure coefficients are shown in Figs 13 and 14.

1. The lateral pressure coefficient is constant. The support of the second test point is comparable with that of the first test point. The anchoring length of the first layer support needs to be lengthened to 0.4 m under hydrostatic stress, 0.3 to 0.7 m when the lateral pressure coefficient is 1.5, and 0.8 to 0.9 m when the lateral pressure coefficient is 2.0 in the direction of the roof, arch shoulder, and sideway, respectively.
2. The anchoring length of the second layer support needs to be lengthened to 1.0 m under hydrostatic stress, 0.7 to 1.2 m when the lateral pressure coefficient is 1.5, and 1.3 to 1.4 m when the lateral pressure coefficient is 2.0 in the direction of the roof, arch shoulder, and sideway, respectively.
3. The cable length of the third layer support needs to be lengthened to 0.9 m under hydrostatic stress, 0.9 to 1.2 m when the lateral pressure coefficient is 1.5, and 1.5 to 1.9 m when the lateral pressure coefficient is 2.0 in the direction of the roof, arch shoulder, and sideway, respectively.

**Fig 15:**
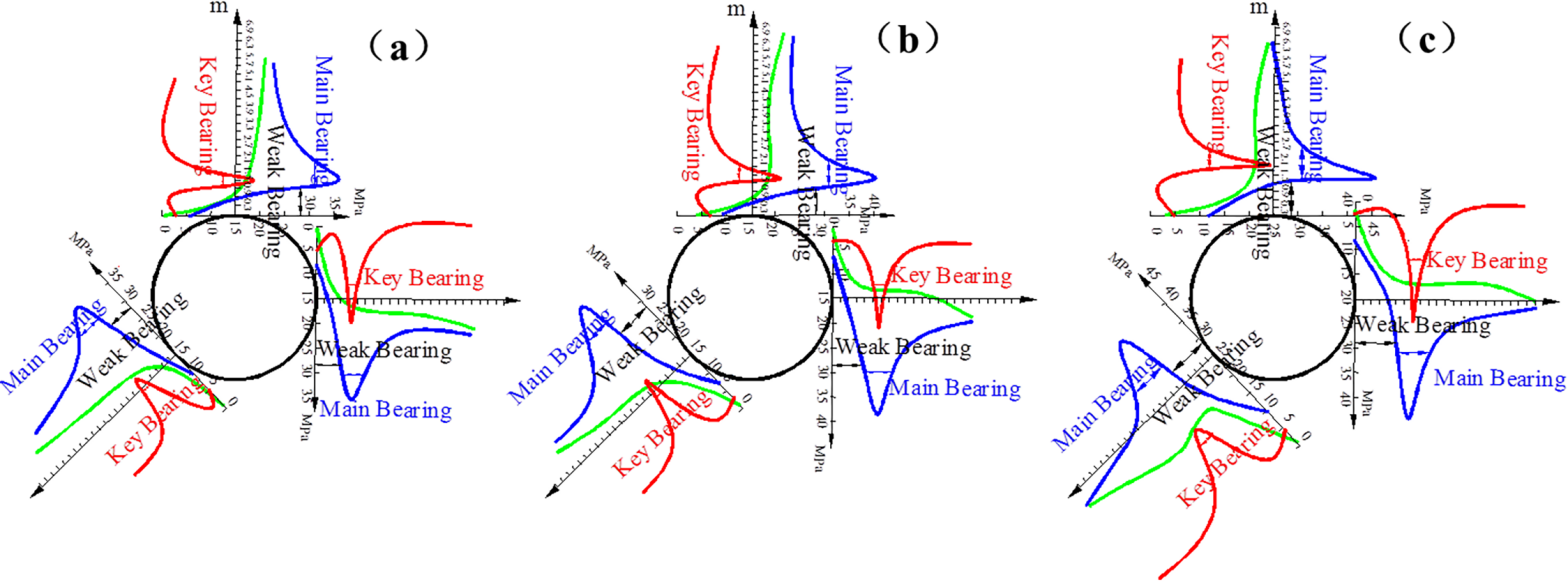
Bearing structure of the cross point 1 under multi-echelon support when the horizontal press coefficient is (a) *λ* = 1; (b) *λ* = 1.5; and (c) *λ* = 2.

**Fig 16:**
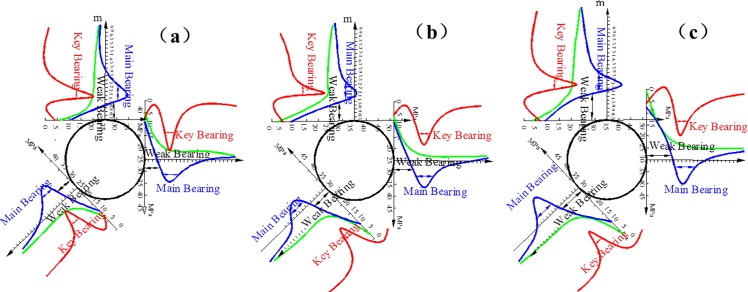
Bearing structure of the cross point 2 under multi-echelon support when the horizontal press coefficient is (a) *λ* = 1; (b) *λ* = 1.5; and (c) *λ* = 2.

The scopes of the bearing structure with multi-echelon support in Figs 15 and 16 are comparable with the scopes without support in Figs 11 and 12. This result clearly indicates that each bearing scope decreases and moves closer to the wall, while the influence of the lateral pressure coefficient on the stability of the bearing structure is not apparent. Multi-echelon support can effectively reduce the thickness of the weak bearing area and restrict the extension range of the main and key bearing areas. The outer boundaries of the weak, key and main bearing areas in the direction of the roof, two sides, and arch foot are listed in Table 5.

**Table 5:** Scope of bearing structure under multi-echelon support.

Cross point	Outer boundary/m	*λ* = 1	*λ* = 1.5			*λ* = 2		
			Roof	Side	Arch foot	Roof	Side	Arch foot
Cross point 1	Weak bearing area	0.90	1.00	1.10	1.20	1.30	1.50	1.60
	Key bearing area	1.40	1.85	2.00	2.10	2.40	2.55	2.70
	Main bearing area	2.00	2.10	2.25	2.40	2.60	2.80	3.10
Cross point 2	Weak bearing area	1.30	1.40	1.5	1.60	1.85	1.90	2.00
	Key bearing area	2.25	2.70	2.80	2.90	3.15	3.20	3.35
	Main bearing area	2.60	3.10	3.15	3.40	3.60	3.70	3.95

**Fig 17:**
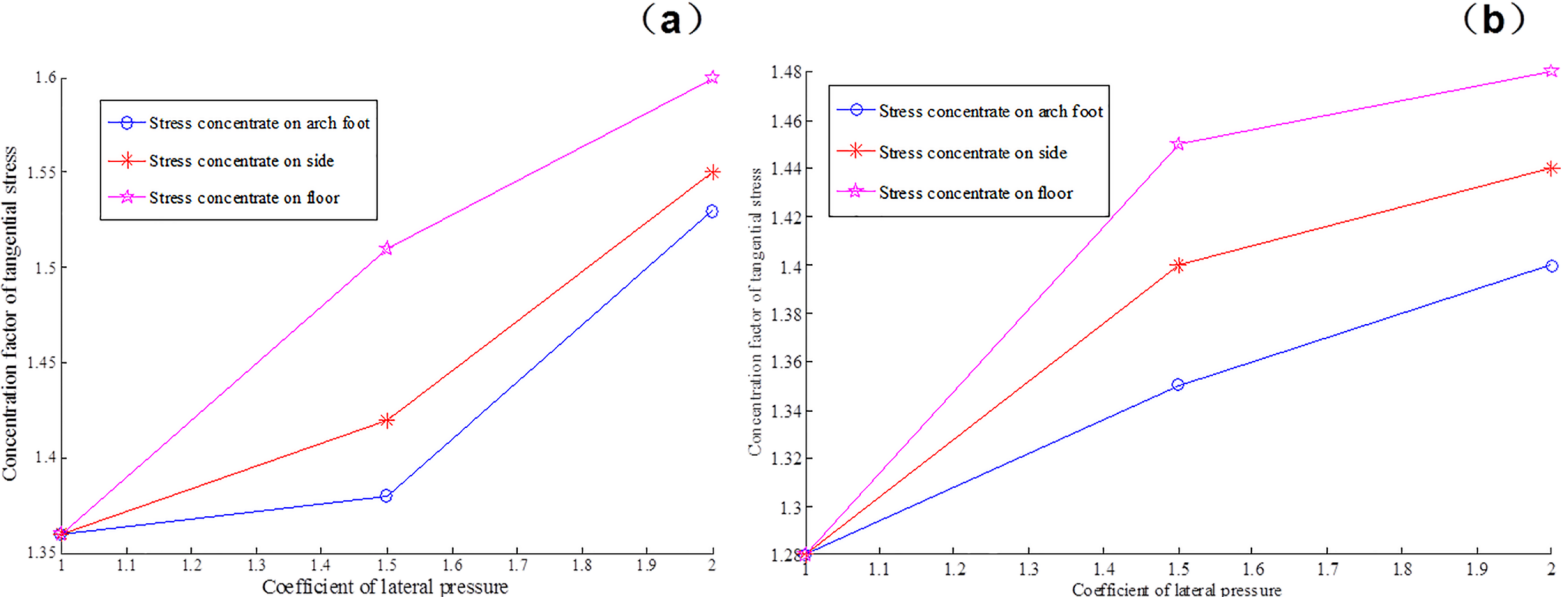
Concentration degree of tangential stress in cross point 1 (a) without support and (b) with multi-echelon support.

**Fig 18:**
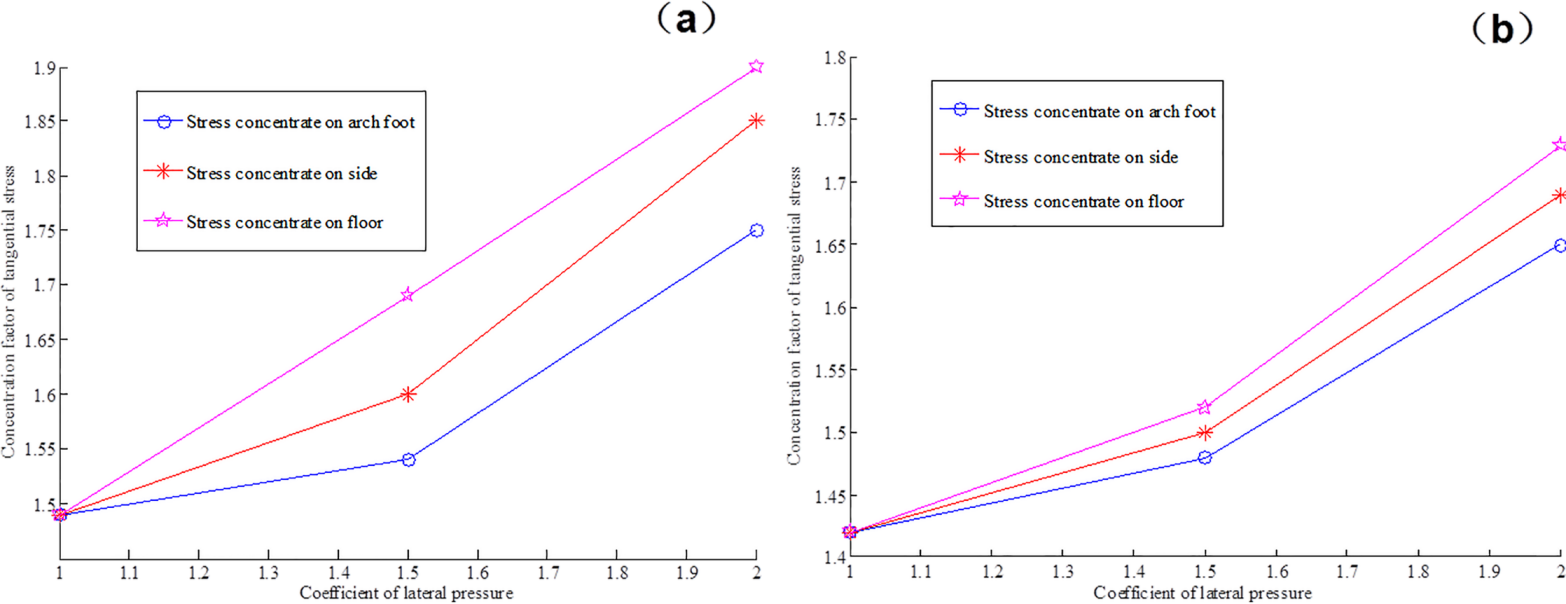
Concentration degree of tangential stress in cross point 2 (a) without support and (b) with multi-echelon support.

Figs 17 and 18 provide the tangential stress concentration factors of the cross points 1 and 2 in the process of excavation and support under different lateral pressure coefficients.

Figs 17 and 18 indicate that the stress concentration coefficients of the cross points 1 and 2 in the direction of the roof, two sides, and arch foot decrease. In turn, the concentration coefficient increases with the increase in lateral pressure coefficient. The degree of stress concentration in the cross point 1 is higher than that in the cross point 2. This condition indicates that stress concentrates easily in the surrounding rock with a high degree of integrity.

### 5.3. Surrounding rock fracture development

**Fig 19:**
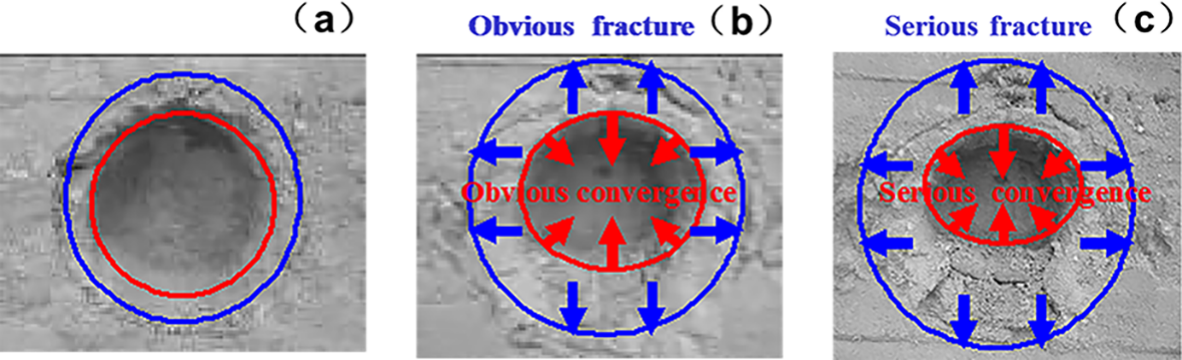
Surrounding rock burst development in cross point 1 without support when horizontal press coefficient is (a) *λ* = 1; (b) *λ* = 1.5; and (c) *λ* = 2.

**Fig 20:**
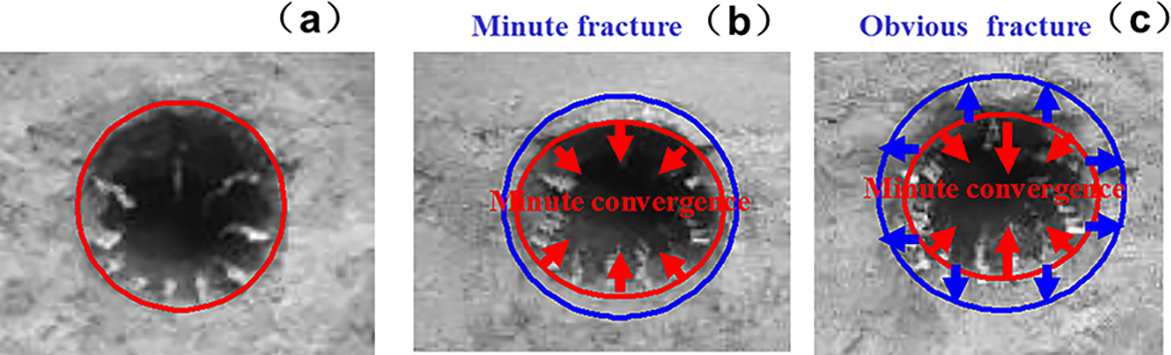
Surrounding rock burst development in cross point 1 with multi-echelon support when horizontal press coefficient is (a) *λ* = 1; (b) *λ* = 1.5; and (c) *λ* = 2.

**Fig 21:**
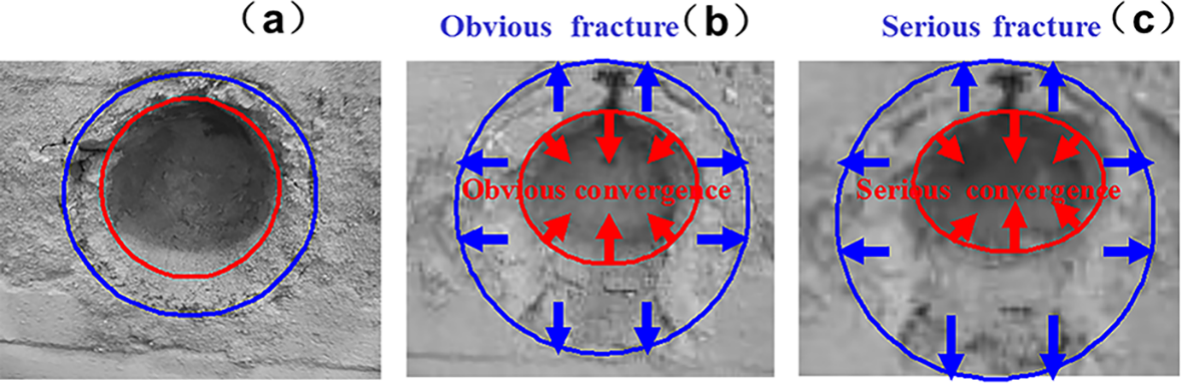
Surrounding rock burst development in cross point 2 without support when horizontal press coefficient is (a) *λ* = 1; (b) *λ* = 1.5; and (c) *λ* = 2.

**Fig 22:**
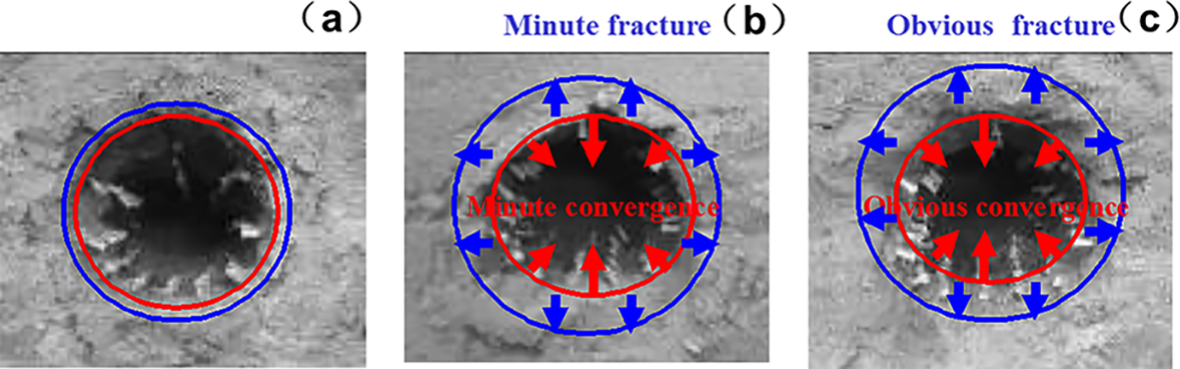
Surrounding rock burst development in cross point 2 with multi-echelon support when horizontal press coefficient is (a) *λ* = 1; (b) *λ* = 1.5; and (c) *λ* = 2.

The fracture development law of deep surrounding rock is determined by observing the rupture development of the roadway under different factors, namely, lithology, lateral pressure coefficient, and support method. The experimental results are listed in Figs 19–22.

The convergence analysis of the roadway cross-section is shown in Figs 19–22. The cross point 2 without support is compared with the cross point 1. The convergence of the roadway cross-section increases with horizontal pressure, whose influence on the fracture development of the surrounding rock is significant. In the same cross point, when lateral pressure coefficient is *λ* = 1, the fracture of the surrounding rock appears circular. When the horizontal stress increases, the convergence values of the roadway cross-section of the floor and roof clearly increase. The circular roadway cross-section tends to be an oval section, with a long horizontal axis and a short vertical axis.

With multi-echelon support, the convergence value of the roadway cross-section decreases obviously, and its shape tends to become circular. The convergence value of the cross-section is high only when *λ* = 2. The sectional convergence values in different cross points, horizontal pressures, and support conditions are listed in Table 6.

**Table 6:** Convergence value of different cross points.

Cross points	Support condition	Convergence value of roadway	*λ =* 1	*λ* = 1.5			*λ* = 2		
				Roof	Arch foot	Sides	Roof	Arch foot	Sides
Cross point 1	Without support	Test value/cm	0.90	1.30	2.70	1.10	2.90	6.30	2.10
		Real value/m	0.23	0.33	0.68	0.28	0.73	1.58	0.53
	Multi-echelon support	Test value/cm	0.60	1.10	1.90	0.80	1.50	3.60	1.40
		Real value/m	0.15	0.28	0.48	0.20	0.38	0.90	0.35
Cross point 2	Without support	Test value/cm	1.30	2.60	3.80	1.40	2.90	7.50	1.80
		Real value/m	0.33	0.65	0.95	0.35	0.73	1.87	0.45
	Multi-echelon support	Test value/cm	0.70	1.20	2.10	1.10	1.60	3.90	1.50
		Real value/m	0.18	0.30	0.53	0.28	0.40	0.98	0.38

The fracture development of the surrounding rock is depicted in Figs 18–21. The cross point 2 without support is compared with the cross point 1. The fracture development of the surrounding rock becomes increasingly evident with increasing horizontal pressure. In the same cross point, horizontal pressure significantly influences the fracture of the surrounding rock. The fractured form is roughly circular. When the lateral pressure coefficient increases, the fracture degree in the direction of the roof, two sides, and floor increases dramatically in turn, and its caving height increases with the occurrence of floor heave and roof wedge caving. The wedge caving of the cross point 2 when the lateral press coefficient is *λ* = 2.

The fracture range decreases under multi-echelon support. Under hydrostatic pressure, cracks only appeared at the cross point 2. When the lateral press coefficient is 1.5 or 2, the range of cracks increases slightly but the roof wedge caving and floor heave are restricted effectively. The fracture range under different support methods, cross points, and lateral pressures is listed in Table 7.

**Table 7:** Range of fracture zones of surrounding rock.

Cross points	Support condition	Fractured range	*λ* = 1	*λ* = 1.5			*λ* = 2		
				Roof	Arch foot	Sides	Roof	Arch foot	Sides
Cross point 1	Without support	Test value/cm	5.80	6.20	10.60	8.50	6.90	12.50	9.80
		Real value/m	1.45	1.55	2.70	2.13	1.73	3.12	2.45
	Multi-echelon support	Test value/cm	3.50	4.50	5.00	4.60	5.60	6.10	5.80
		Real value/m	0.88	1.13	1.25	1.15	1.40	1.53	1.45
Cross point 2	Without support	Test value/cm	8.20	8.90	14.5	10.2	10.8	16.0	12.3
		Real value/m	2.10	2.23	3.63	2.55	2.70	4.00	3.07
	Multi-echelon support	Test value/cm	5.40	6.70	7.00	6.80	8.40	8.70	8.50
		Real value/m	1.40	1.68	1.75	1.70	2.10	2.18	2.13

## 6. Research on effect of multi-echelon support

### 6.1 Industrial test on loose circle

Deep-hole observation stations are placed at each cross point during the roadway excavation and supporting.

**Fig 23:**
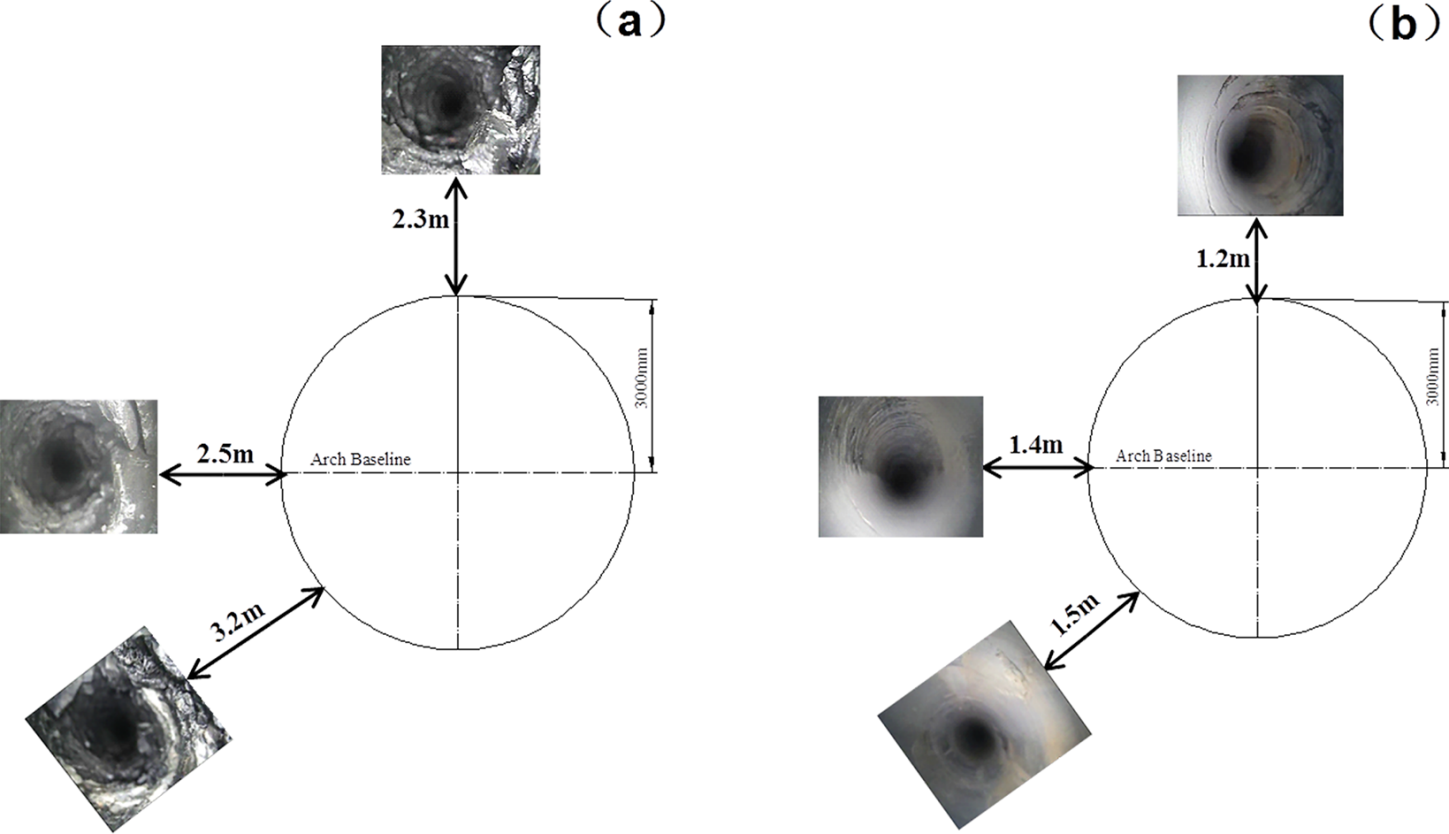
Loosing circle in cross point 1 (a) without support; (b) with multi-echelon support.

**Fig 24:**
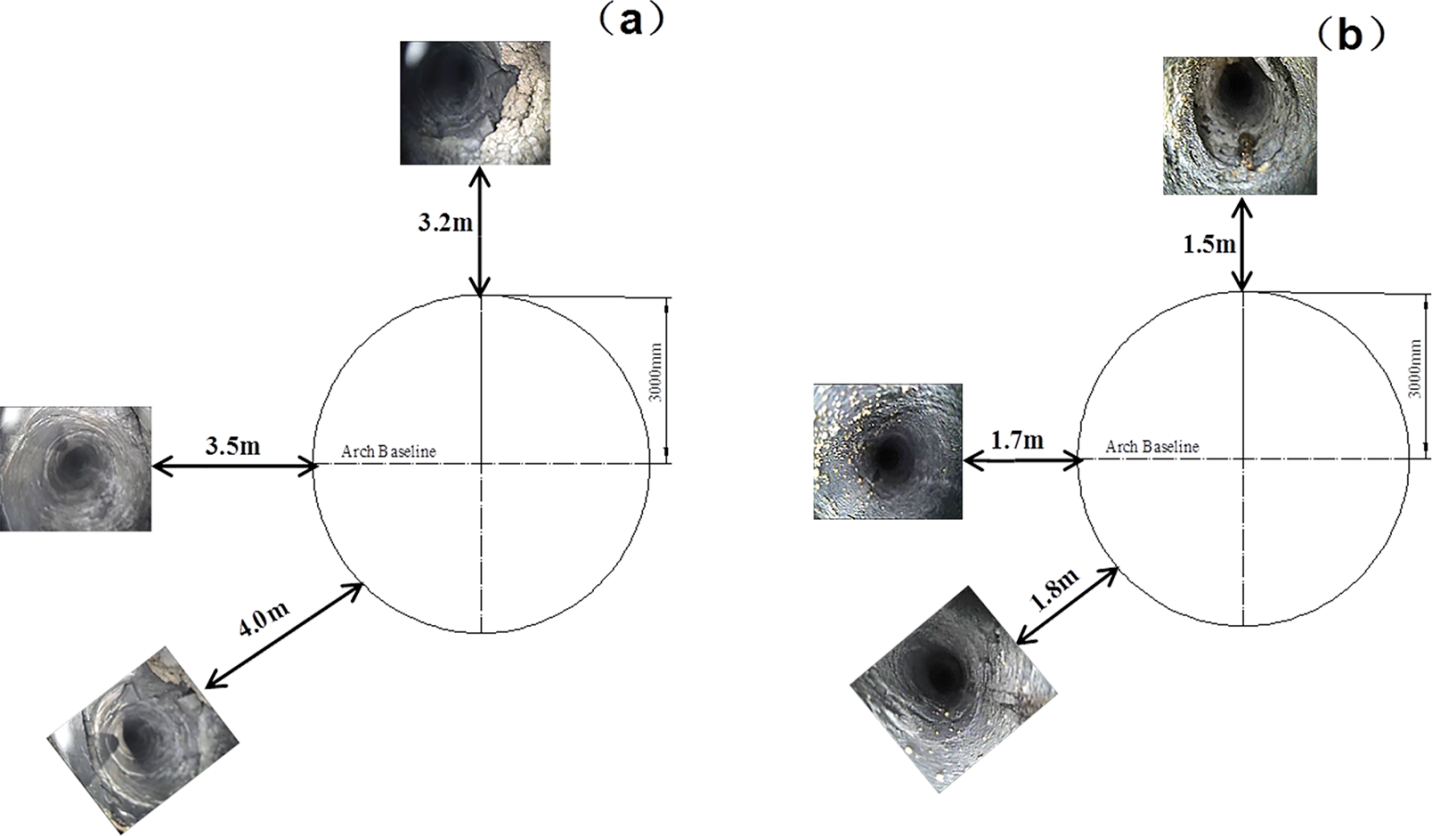
Loosing circle in cross point 2 (a) without support; (b) with multi-echelon support.

The deep-holes in cross points 1 and 2 lie along the direction of roof, two sides and floor. The datum shown in Figs 23 and 24 are obtained by the observation.

Fig 23 indicates that the fracture ranges of the cross point 1 in the direction of the roof, side, and bottom arch foot are 2.3, 2.5, and 3.2 m, respectively. With the multi-echelon support, the fracture ranges in the direction of the roof, side, and bottom arch foot decrease to 1.2, 1.4, and 1.5 m, respectively. In summary, the brittle rupture failure without support tends to cause a few fractures in a roadway with multi-echelon support.

Fig 24 shows that the fracture ranges of the cross point 2 in the direction of the roof, side, and bottom arch foot are 3.2, 3.5, and 4.0 m, respectively. With multi-echelon support, the fracture ranges in the direction of the roof, side, and bottom arch foot decrease to 1.5, 1.7, and 1.8 m, respectively. In summary, the soft dilatation failure without support tends to cause brittle rupture characteristics of hard rock failure with multi-echelon support.

### 6.2 Numerical simulation of loose circle

**Fig 25:**
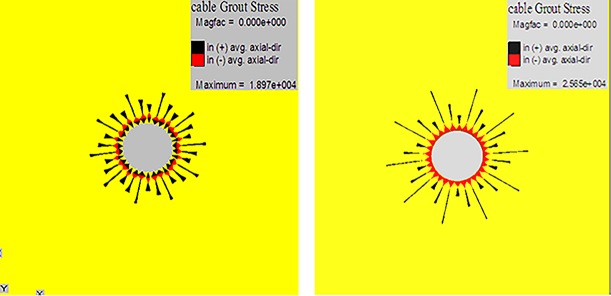
Grouting effect of multi-echelon support in (a) cross point 1; (b) cross point 2.

By studying the histograms of the rock strata near cross points 1 and 2 and utilizing computer-aided design to draw the pictures with a 1:1 scale, roadway models can be established by using the FLAC3D. The models are designed as strain-softening models based on the Mohr–Coulomb criterion. Specifically, the boundary conditions are designed as follows. The sides of the models are constrained in horizontal movement. The bottoms of the models are constrained in vertical movement. Multi-echelon support advanced with the excavation during the process. The grouting effect of multi-echelon support in different cross points is shown in Fig 25. With the advance of excavation and support, we analyzed the impact of plastic zone distribution.

**Fig 26:**
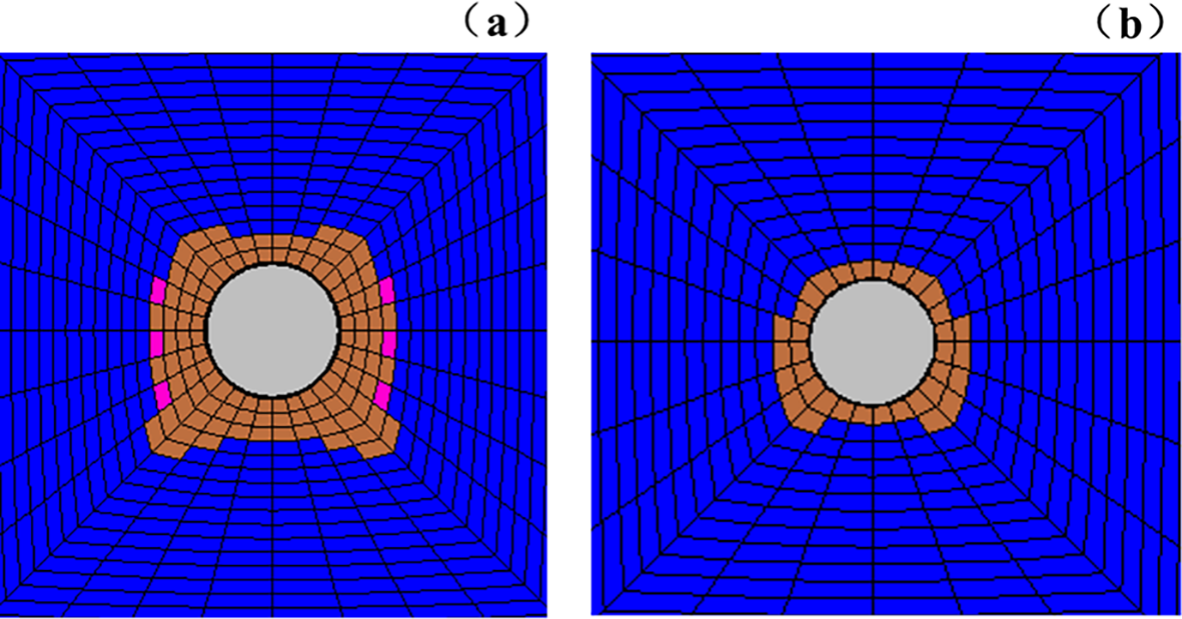
Loosing circle in cross point 1 (a) without support; (b) with multi-echelon support.

Fig 26 indicates that the fracture ranges of the cross point 1 in the direction of the roof, side, and bottom arch foot are 2.0, 3.5, and 4.5m, respectively. With multi-echelon support, the fracture ranges in the direction of the roof, side, and bottom arch foot decrease to 1.0, 2.0, and 2.0 m, respectively.

**Fig 27:**
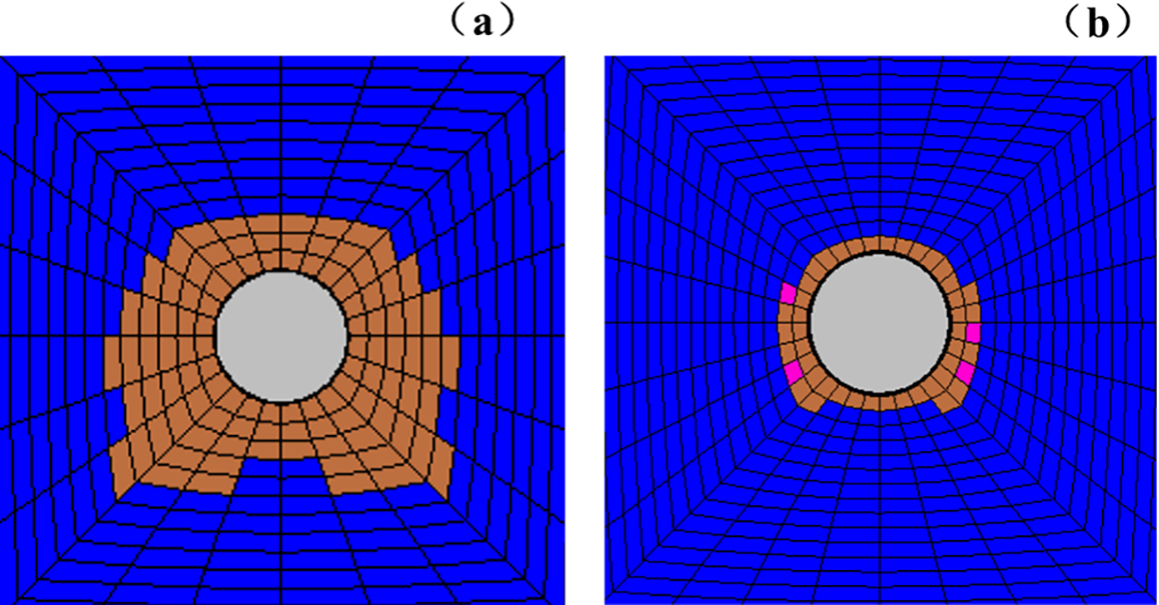
Loosing circle in cross point 2 (a) without support; (b) with multi-echelon support.

Fig 27 shows that the fracture ranges of the cross point 2 in the direction of the roof, side, and bottom arch foot are 3.0, 4.5, and 5.5 m, respectively. With multi-echelon support, the fractured ranges in the direction of the roof, side, and bottom arch foot decrease to 1.0, 1.5, and 2.0m, respectively.

## 7. Conclusions

The following conclusions can be drawn from the current study:

1. The influence of lithology and lateral press on the structural stability of roadway bearings is analysed. Lateral press influences the structural stability of soft roadway bearings with significant depth. As a result, the weak bearing area enlarges the range, and the main and key bearings area transfer externally. The instability of hard roadway bearing structure increases in the direction of the roof, two sides, and bottom arch foot. The thickness values of the weak bearing areas in the cross points 1 and 2 are 0.80*r*_0_ and 1.10*r*_0_, respectively. The expansion ranges of the key bearing area are 1.13*r*_0_ and 1.60*r*_0_, and the expansion ranges of the main bearing area are 1.22*r*_0_ and 1.97*r*_0_, respectively.
2. The influence of multi-echelon support on the structural stability of roadway bearings is examined. The maximum supporting ranges of hard roadways and soft roadways in the direction of the arch shoulder are 4.4 and 6.3 m, respectively. With multi-echelon support, the adverse influence of high horizontal stress on the stability of deep roadways is lessened. The maximum shrinking ranges of the weak bearing area are 0.8 and 1.3 m, respectively. The expanded area and substantially transferred ranges of the key and main bearing areas in the soft roadways are restricted obviously. According to the concentration of secondary stress, the bearing capacity is stronger after roadway excavation, and the bearing capacity improves obviously with multi-echelon support.
3. The fracture development law of surrounding rock is analysed. Without support, the convergence values of two sides increase evidently with horizontal pressure. As a result, the circular section of the roadway turns into an oval section. The expansion range and degree aggravation of the fracture area around the floor is evident. Floor heave and wedge roof caving worsen. Multi-echelon support decreases the adverse influence of high horizontal stress on the convergence value of the roadway cross section. It also restricts the damage of the surrounding rock and prevents floor heave and wedge roof caving. According to the in-situ test and numerical simulation, multi-echelon support can restrict the malignant expansion of the damage range and its degree of aggravation.
