# Correction: Experimental research on the structural instability mechanism and the effect of multi-echelon support of deep roadways in a kilometre-deep well

**DOI:** 10.1371/journal.pone.0199490

**Published:** 2018-06-18

**Authors:** Rui Peng, Xiangrui Meng, Guangming Zhao, Yingming Li, Jianming Zhu

The following information is missing from the Funding section: This research is supported by the Science and Technology Research for Heibei University (Grant no. QN2018302) to RP.
