# Correction: Experimental research on the structural instability mechanism and the effect of multi-echelon support of deep roadways in a kilometre-deep well

**DOI:** 10.1371/journal.pone.0204059

**Published:** 2018-09-11

**Authors:** Rui Peng, Xiangrui Meng, Guangming Zhao, Yingming Li, Jianming Zhu

The following information is missing from the Funding section: This research is supported by the National Natural Science Foundation of China (grant 51804119) to RP.
